# Seroprevalence of measles vaccine antibody response in vertically HIV-infected children, in Morocco

**DOI:** 10.1186/s12879-018-3590-y

**Published:** 2018-12-19

**Authors:** Houda Haban, Soumia Benchekroun, Mina Sadeq, Latifa Tajounte, Hinda Jama Ahmed, Abdelaziz Benjouad, Said Amzazi, Hicham Oumzil, Elmir Elharti

**Affiliations:** 1 Department of Virology, National Reference Laboratory for HIV, National Institute of Hygiene, Rabat, Morocco; 2 Immunology-Biochemistry Laboratory, Faculty of Sciences, University Mohamed Vth, Rabat, Morocco; 3 Pediatric Infectious Disease Clinic, Ibn Sina University Hospital, Rabat, Morocco; 4 Environmental Epidemiology Unit, National Institute of Hygiene, Rabat, Morocco; 5 Department of Virology, National Reference Laboratory for Measles, National Institute of Hygiene, Rabat, Morocco; 6 Faculty of Medicine, Eastern Africa University, Bosaso, Somalia; 7 International University of Rabat, Salé, Morocco

**Keywords:** HIV-infection, Measles prevention, Seroprotective response, HAART therapy

## Abstract

**Background:**

The widespread use of an effective and safe vaccine to measles has substantially decreased morbidity and mortality from this epidemic. Nevertheless, HIV-infected children vaccinated against measles may develop an impaired vaccine response and remain susceptible to this disease. In Morocco, infants are routinely vaccinated against measles, regardless of their HIV serostatus. An evaluation of the immunization of these children may be of paramount importance to implement timely measures aimed at preventing measles transmission.

**Methods:**

In this study, we have enrolled 114 children vaccinated against measles, 50 children prenatally infected with HIV and 64 HIV-uninfected children. For all children, blood samples were taken to measure anti-measles IgG by EIA and CD4 count by flow cytometry. Additionally, HIV viral load was determined by automated real time PCR, for HIV-infected children.

**Results:**

The seroprotective rate of IgG anti-measles antibodies was significantly lower among HIV-infected children (26%) compared with HIV-uninfected children (73%) (*p* < 0.001). Within HIV-infected children group, the comparison of variables between children without seroprotective seroconversion to measles and those with seroprotective immunity, displayed that sex and age were not statistically different, *p* > 0.999 and *p* = 0.730, respectively. However, CD4 count was lower among children with negative serostatus to measles (23% versus 32%, *p* < 0.001). Furthermore, viral load was higher, with 2.91 log_10_ ± 2.24 versus 1.7 log_10_ ± 1.5 (*p* = 0.042). Finally, 62% of children with a negative vaccine response to measles were under HAART therapy, versus 92% (*p* = 0.008).

**Conclusion:**

The majority of HIV-infected children vaccinated against measles develop a suboptimal seroprotective titer, and therefore remain at risk for this highly infectious disease. These data in combination with international recommendations, including recent WHO guidance on vaccination of HIV-infected children, suggest there is a need for national measures to prevent these children from measles.

## Background

Measles are highly contagious paramyxoviruses, which cause substantial mortality among children and even adult. Luckily, this disease is vaccine preventable, and the widespread use of effective and affordable vaccine has brought about a significant drop of the burden of measles epidemic [1–4]. For instance, it is estimated that the mass vaccination has saved at least 20 million lives, between 2000 and 2015 [5]. Given the impact of vaccination programs all over the world, WHO has established goals aimed at eliminating measles by 2015 or before [6]. Until now, the region of the Americas was the first in the world to have eliminated measles, in 2016, after its interruption in 2003. This achievement was a culmination of many years of sustained efforts, involving widespread vaccination implementation [7]. On the other hand, the other regions in the world are still struggling to attain this objective. For example, within the Eastern Mediterranean Region (EMR), measles remain a public health challenge, despite efforts made so far. Actually, there was a major increase of measles cases from 12,186 in 2008 to 36,456, in 2012. In 2015, the number of reported measles cases was 21,335. This epidemiological situation was attributed to important outbreaks in countries with political instability and conflicts, mainly countries that have experienced the “Arab Spring” [5, 8]. Consequently, the WHO goal to eliminate measles in this region, was deferred to 2020 [9].

Morocco, an EMR country, has been followed the WHO recommendations to fight and eliminate the measles epidemic. In this context, monovalent measles vaccine has been incorporated by the Moroccan ministry of health, through the expanded program on immunization (EPI), since 1981. Since then, all infants have been vaccinated at 9 months, according to the national vaccine schedule. In 1984, the vaccination was expanded throughout the country. As a result, a significant decrease of the spread of epidemic was accordingly notified, and the annual cases number decreased from 2574 in 1997 to 720 in 2012 [10, 11]. To limit further measles spread, the national program of immunization (formerly known as EPI), introduced the second dose of measles vaccine through national campaigns, in 2008 and 2013. In 2008, measles vaccine campaign targeted children aged between 9 months to 15 years, by using monovalent vaccine, and young women of childbearing, by providing bivalent vaccine (measles-rubella). In 2013, the vaccination campaign focused on children and young adults aged 9 months to 19 years, using the bivalent vaccine. Following these measures, the measles vaccine-containing-first-dose coverage has been gradually increased, to reach 96% in 2010. Finally, to maximize measles protection, the second dose of bivalent vaccine was introduced into routine vaccination, in 2014 [12, 13].

Measles vaccination of HIV-infected children is crucial for their protection, since they are more susceptible to measles than HIV-uninfected children. Besides, measles coinfection can be more serious in immunocompromised children [14–16]. Nonetheless, the vaccination of HIV-infected children against measles might lead to an impaired vaccine response [17, 18]. Additionally, the vaccine-induced immunity is likely to shrink more rapidly in children with HIV than those without HIV. Therefore, these patients may remain vulnerable to this serious infection [19, 20]. In this framework, international guidelines were developed to protect these patients from coinfection with measles. Basically, the recommendations consist of three key components. Firstly, immunocompromised HIV-infected children should not be vaccinated as long as CD4 count is less than 15%, since the vaccine is a life-attenuated virus. Secondly, children without evidence of severe immunosuppression i.e. their CD4 count is more than 15%, should be vaccinated against measles. Finally, vaccinated subjects under HAART therapy should be monitored for assessing the serological response to identify those who lack evidence of measles immunity, in order to revaccinate them [21–23].

Despite the important efforts deployed by the Moroccan ministry of health, towards measles control and elimination, until now there is no national guidelines which focus on optimizing the immunization of HIV-infected children against measles, and all infants are still vaccinated, without consideration of whether they are HIV-infected or not. Furthermore, data relevant to seroprotective response rate to measles, for this category of children, are missing. Such data are of capital importance to guide vaccine policy against measles.

We therefore, conducted this study in order to assess and compare the immune response to measles vaccine among HIV-infected children and children without HIV.

## Methods

This study was conducted in Pediatric Infectious Disease Clinic, Ibn Sina University Hospital, in Rabat, the capital of Morocco. The enrollment involved 114 children, 50 children infected with HIV (HIV group) and 64 HIV uninfected children (control group). The HIV group represented children infected with HIV, via mother-to-child transmission, recruited in this study, during their routine clinic visit for the management of HIV infection; from 13 January 2011 to 16 June 2013. Subjects from the control group, were children visiting the same Pediatric Infectious Diseases Clinic, during the same period, for respiratory diseases management and screened negative for HIV, by rapid test (HIV Determine, Alere). All recruited children from the HIV group and the control group were aged between 10 months and 10 years, received the measles vaccine according to the national program of immunization, as proved by the vaccination card, and did not present any primary immunodeficiency disease. HIV-infected children and controls were matched by age.

Prior to any enrollment in this study, parents or guardians of children signed a written informed consent. In addition, the study received the clearance of the Ethic Committee of Biomedical Research, Medical School and Pharmacy, University Mohammed V^th^, Rabat, Morocco. After the enrollment, peripheral blood was collected by venipuncture and a questionnaire was administered to child’s caregiver to collect demographic and clinical data. All laboratory tests were performed in National Reference Laboratory for HIV, Department of Virology, National Institute of Hygiene, in Rabat.

### Laboratory tests

For all samples, anti-measles antibodies quantification were performed by using a commercial enzyme immunoassay (EIA) test that detect measles IgG antibodies (Enzygnost, Siemens, Germany). According to the manufactures’ interpretation, samples that gave delta OD values > 0.2 are considered positive. Those with delta OD < 0.1 are supposed negative. Specimen with 0.1 < delta OD < 0.2 are considered equivocal. The antibody titer was calculated in mIU/ml as described in the package insert, and therefore measles IgG seronegative response was defined for titer < 150, equivocal response for 150 ≤ titer < 304 and the seroprotective response was defined as antibody titer ≥304.

CD4 count were determined by flow cytometry, for both groups. Briefly, the whole blood was immunostained by using tritest CD3 FTTC/CD4 PE/CD45 PerCP (Becton-Dickinson, USA). Then the red cells were lysed with FACS/lyse solution (Becton-Dickinson, USA). Finally, the samples acquisition and analysis were performed on flow cytometry, using CellQuest Pro software (FacsCalibur, Becton-Dickinson, USA) to generate CD4%.

HIV Viral load was determined for the HIV group, using a fully automated real time PCR testing system (Abbott real Time HIV-1, USA). Obtained values in copies/ml of plasma were log_10_ transformed.

### Statistical analysis

We explored data on age, gender, CD4 count, and anti-measles antibodies in HIV-group as well as in controls and we represented calculated descriptive statistics in graphics. In the HIV-infected group, we also examined data on HIV viral load and compared the proportion of HAART therapy to an expected proportion (0.5). The Clopper-Pearson method is used for the exact confidence interval. The exact p hypothesis test is provided for the proportion in comparison with the expected proportion i.e. 0.5.

The variables cited above were also compared for measles seroprotective response and measles seronegative response, among HIV-infected children. The equivocal results were considered negative in this analysis. The method of Miettinen and Nurminen was used to construct the confidence interval for the difference between the proportions: proportion difference and an approximate two sided p were given. We compared two independent groups using Mann Whitney test, a two sided *p*-value was given and a confidence intervals was constructed for the difference between the medians. Medians or proportion difference were considered statistically significant at *p* < 0.05.

We used StatsDirect statistical software version 3.0.194 (StatsDirect Ltd., Cheshire, UK) for statistical analyses.

## Results

**Fig. 1:**
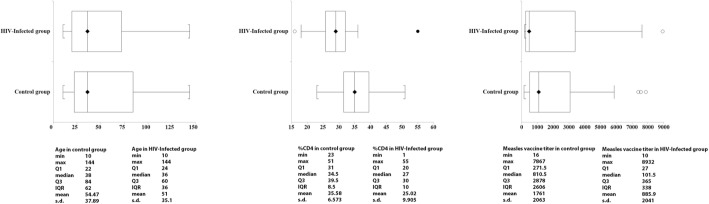
Descriptive analysis of studied variables in children infected with HIV (HIV group) and children without HIV (control group)

We studied the anti-measles response in 50 HIV-infected and 64 uninfected children. A proportion of 38% were male in the HIV-infected group, 58% in the control group. Figure 1 and Table 1 depict descriptive statistics in both groups. There was no difference means (medians) in age between the two groups. The mean age was 51 months in HIV-infected children, 54 months in controls. A statistically significant differences in gender proportion (*p* = 0,03) and in CD4% (*p* < 0,0001) were seen. In the HIV-infected children, the proportion of children under HAART therapy was statistically significantly different from 0.5 (*p* = 0,007 and 95% CI = [55 to 82%]). The antibody level in HIV patients is lower when compared to control group, with a median of 101 IU/ml (IQR 27–365 IU/ml) versus 810 IU/ml (IQR 271–2878 IU/ml).

**Table 1:** Demographic and clinicobiological data among HIV-infected children group

Variable	Seronegative measles antibody, *n* = 37	Seropositive measles antibody, *n* = 13	*p* value
Age (months)	50 ± 31	55 ± 47	0.729
Female	62%	61.5%	> 0.9999
CD4%	23	32	0.0005
HAART therapy	62%	92%	0.0081
Viral load (log_10_)	2.91 ± 2.24	1.07 ± 1.5	0.043

Table 2 shows that the seroprotective rate for Measles was significantly lower in HIV-infected children than in HIV-uninfected children, 26% versus 73% (*p* < 0.0001). Equivocals were not statistically different in the two compared groups (*p* = 0.62).

**Table 2:** Variables among HIV-infected children with seronegative and seropositive measles vaccine response

Measles serostatus	Negatives, %(n)	Equivocals, %(n)	Positives, %(n)
HIV-infected children	58% (28)	16% (7)	26% (15)
Control group	12.5% (8)	14.1% (8)	73.4% (48)
*p* value	< 0.0001	0.62	< 0.0001

Within HIV-infected children vaccinated to measles (Table 1), the comparison of variables among children without suboptimal seroconversion and children with seroprotective antibody level, shows that both sex and age and are not different in both groups, *p* > 0.9999 and *p* = 0.73, respectively. However, HIV-infected children with negative measles IgG have lower CD4% (23% versus 32%, *p* = 0.0005) and tend to have higher HIV viral load (2.91 ± 2.24 log_10_ versus 1.7 ± 1, 5 log_10_, *p* = 0.04). Finally, the percent of children that are under HAART therapy is lower among HIV-infected children with a negative seroconversion to measles (62% versus 92%, *p* = 0.008).

## Discussion

Measles vaccine is known to produce protective levels of antibodies in more than 90% among immunocompetent children [24, 25]. However, when it comes to HIV-infected children, low immunogenicity and ongoing decay of measles antibodies are the hallmark of this vaccine response [26, 27].

This report represents the first evaluation of measles vaccine response, in a group of vertically HIV-infected children, and children that are not infected with HIV, in Morocco. The study demonstrates that HIV-infected children are less likely to develop a protective seroconversion, in spite of measles vaccination. In fact, in the HIV children group, the prevalence of a seroprotective response is only 26%, whereas it is around 73% among the control group (*p* < 0.001). In other words, most of these vaccinated HIV-infected children may remain at risk for contracting measles. Our findings concord with others studies that have reported a poor serological response, ranging from 23 to 33% [28, 29]. Furthermore, the level of antibody to measles in HIV patients, found in this study is lower when compared to control group. In fact, it was reported that the magnitude of the measles vaccine response in children with HIV infection tends to diminish and even IgG antibodies can revert to a negative serostatus [29]. This low responsiveness to measles vaccine, in our study, may be explained by a failure in the production of antibodies following the primary vaccination or antibodies loss, over time, after initial induction. The wide age range of time (10 months to 10 years), may also account for antibody waning [30, 31]. Additionally, immunosuppression state of HIV-infected children is reported to lead to such poor vaccine responsiveness [32]. Nevertheless, our data suggest that studied patients are not immunocompromised.

The comparison of measles vaccine response in HIV-infected subjects, in this study, reveals that 92% of HIV-children with protective antibody titer were under HAART treatment, versus 62% for children with a seronegative vaccine response (*p* = 0.0081). Moreover, children with optimal response, tend to have a higher CD4% and a lower viral load. In fact, it is established that the replication of the HIV virus is detrimental to the immune system, through CD4 + T cells depletion, and hence affects the humoral immunity [33–35]. Fortunately, since the advent of HAART treatment, this trend has been reversed by an effective suppression of viral multiplication and subsequent immune system reconstruction. In this study, despite of the institution of HAART therapy in 70% of HIV-infected patients, the immune response to measles remains impaired. It is worthwhile noting that we do not know the timing of antiretroviral therapy, nor its duration, though we can speculate that this result may be due to the fact that these children are still at the beginning of the treatment and then the impact of treatment is not yet effective. In addition, slow humoral immunity restoration may account for this result [36]. Another possible explanation of this measles vaccine low responsiveness might be a late initiation of HAART therapy, i.e. after the first year of life [37]. However, others studies have reported that despite the immuno-virological effectiveness of antiretroviral treatment, humoral immunity acquired after the primary vaccination may not be fully restored, as defect in CD4 functionality may still exist. [26, 31]. Consequently, these data reinforce the concept that HAART therapy is necessary for immune reconstruction, but it is not sufficient to reliably recover vaccine-induced immunity. Indeed, a study conducted on HIV-children under potent HAART therapy, has revealed that seroprotected children represent only 52%, despite primary vaccination against measles. Furthermore, when non immunized children were revaccinated to measles, around 90% developed a seroprotective humoral immunity [38]. Thus, the best measles immunization outcome requires revaccination of these children once the viral replication is suppressed and the immunity is restored by HAART therapy [39, 40]. In this context, international guidelines have been provided to recommend the revaccination of children after potent HAART treatment [21, 40, 41]. When we take into account all these data, we think that HIV-infected children, who experienced an impaired humoral immunity, need another measles vaccination; to achieve an adequate and sustainable protection to measles.

The findings of the present study are subject to some limitations. Firstly, data related to duration of HAART therapy and its impact on immune restoration were not reported. Secondly, CD4 count, viral replication suppression and HAART institution were not known at time of vaccination. Thirdly, the antibodies against measles measured in this study by EIA, were presumed to be triggered by the vaccine response, nevertheless, they may have resulted from the wild type virus. However, there was no measles outbreak reported, in that period, which might have affected the children of this study. Fourthly, there is a high number of equivocal humoral response to measles. Fifthly, we cannot rule out biases when recruiting HIV-infected children and controls, during this study. Finally, when compared to the control group, the HIV-infected children had less male, in this study.

The present work underscores a lack of immunity to measles among most of vaccinated HIV-infected children. Thus, it is obvious that these children remain predisposed to this disease. These findings are in line with other studies and guidelines that recommend the assessment of measles vaccine response among HIV-infected children, in order to optimize their immunization against this highly infectious disease.

## Conclusions

This study demonstrates that the majority of HIV-infected children vaccinated against measles, fail to develop the protective antibody titer and therefore remain susceptible to this infectious virus. These data in combination with international recommendations including those of WHO, suggest that national measures aimed at protecting these children from measles, should be elaborated.

## Abbreviations

EIA: Enzyme immunoassay
EMR: Eastern Mediterranean Region
EPI: Expanded program on immunization
